# Serum Ferritin and Non-alcoholic Fatty Liver Disease: A Meta-analysis and Systematic Review

**DOI:** 10.5152/tjg.2023.22453

**Published:** 2023-09-01

**Authors:** Junxin Yan, Tongjuan Guan, Meiqi Guo, Jingfang Liu

**Affiliations:** 1 The First Clinical Medical College, Lanzhou University, Lanzhou, Gansu, China; 2 Department of Endocrinology, The First Hospital of Lanzhou University, Lanzhou, Gansu, China

**Keywords:** Ferritin, non-alcoholic fatty liver disease, non-alcoholic steatohepatitis, meta-analysis

## Abstract

**Background/Aims:**

Previous studies have shown that hyperferritinemia is a common phenomenon in non-alcoholic fatty liver disease patients. We aim to further explore the relationship between serum ferritin levels and non-alcoholic fatty liver disease using a meta-analysis.

**Materials and Methods:**

Four Library databases were electronically searched from inception until December 2021 to find prospective cohort or case–control studies examining the relationship between serum ferritin levels and non-alcoholic fatty liver disease, and all kinds of literature were screened according to the inclusion and exclusion criteria. The odds ratio and other related data were extracted, and a meta-analysis was performed.

**Results:**

Eleven studies examining the relationship between serum ferritin levels and non-alcoholic fatty liver disease were included. The serum ferritin levels in the non-alcoholic fatty liver disease group were significantly higher than those without non-alcoholic fatty liver disease group (1.54 ng/mL, 95% CI: 0.85-2.23,* P *< .001). Serum ferritin levels were significantly associated with the risk of non-alcoholic fatty liver disease in both men and women (odds ratio = 2.36, 95% CI: 1.41-3.93, *P* = .001 and odds ratio = 2.93, 95% CI: 1.83-4.69, *P* < .001, respectively), and after adjusting for the parameters, the relationships were still shown to be significant in men and women (odds ratio = 2.24, 95% CI: 1.64-3.05, *P *< .001 and odds ratio = 3.30, 95% CI: 2.13-5.11, *P* < .001, respectively).

**Conclusion:**

Serum ferritin levels were higher in patients with non-alcoholic fatty liver disease than in those without non-alcoholic fatty liver disease and were associated with the risk of non-alcoholic fatty liver disease in both men and women.

**Main Points**

- Serum ferritin levels were higher in patients with non-alcoholic fatty liver disease (NAFLD) than in those without NAFLD.
- Serum ferritin levels were associated with the risk of NAFLD in both men and women.
- Serum ferritin levels were not associated with the risk of non-alcoholic steatohepatitis.

## Introduction

Non-alcoholic fatty liver disease (NAFLD) is defined as excessive liver fat accumulation, with more than 5% of hepatocytes containing triglycerides, free fatty acids, ceramides, and free cholesterol^1^ in the absence of heavy alcohol consumption or medication to induce liver steatosis.^2^ The global prevalence of NAFLD is approximately 25.24%, and NAFLD has gradually become the common cause of morbidity and mortality in patients with liver-related chronic diseases.^3^ In recent years, a considerable amount of evidence has shown the complex interactions between NAFLD and type 2 diabetes and obesity, and NAFLD is also common in people with various diseases, including extrahepatic malignancy, chronic kidney disease, some endocrine diseases (including polycystic ovary syndrome and osteoporosis), brain aging, and cognitive impairment.^4^ Therefore, NAFLD has a significant impact on human health, and the early diagnosis and prevention of NAFLD are of utmost importance.

Non-alcoholic fatty liver disease includes a wide range of histological changes, mainly simple steatosis and non-alcoholic steatohepatitis (NASH).^5^ Steatosis can occur when the amount of fat in the liver cells is ≥5%, and the NASH can be diagnosed when any degree of hepatic steatosis, hepatocellular ballooning, or lobular inflammation occurs.^6^ As NASH progresses, the liver becomes rigid, and liver function declines, leading to cirrhosis, hepatocellular carcinoma (HCC), death, or liver transplantation.^6^ The NASH is the active period of NAFLD, and so we divide NAFLD into NASH and no-NASH. The global prevalence of NASH is 3%-5%,^7^ with approximately 10%-30% of NAFLD patients developing NASH.^8^ Compared with other liver diseases, 5%-50% of patients with HCC present with NASH before developing cirrhosis and routine safety screening, and these tumors tend to be larger and less treatable.^9^

Iron is one of the most important micronutrients in living organisms and is an essential element for respiration and energy production in the mitochondria. Iron may cause oxidative damage to biomacromolecules by promoting the production of intracellular reactive oxygen species and changing the intracellular redox environment, eventually leading to chronic liver disease.^10^ The liver plays a central role in iron metabolism and is the main source of hepcidin, a peptide hormone that regulates iron homeostasis. Excessive iron deposition, hepatocyte necrosis, and systemic inflammation caused by NAFLD may induce the overexpression of hepcidin, which decreases the intestinal iron absorption and increases the iron retention in macrophages and hepatocytes. Ferritin is the primary storage mode of iron in the liver; once iron accumulates in the liver, it may damage the liver cell proteins and DNA through peroxidation stress. Ferritin can directly activate hepatic stellate cells and induce hepatic fibrosis through the nuclear factor κB cascade reaction.^11^

The golden standard for diagnoses of NAFLD is liver pathological tissue biopsy; however, it cannot be used as a routine screening method because of its invasive nature. Therefore, exploring a non-invasive, simple, and economical method for diagnosing NAFLD is a new direction of current research; previous studies have found that hyperferritinemia, with or without increased iron concentration in the liver, is a common manifestation of NAFLD.^12^ One study has showed that serum ferritin levels are correlated with NAFLD occurrence in Korean men.^13^ In this study, the correlation between serum ferritin levels and the risk of NAFLD was analyzed using a meta-analysis to provide new ideas for the early diagnosis of NAFLD.

### MATERIALS AND METHODS

Literature Retrieval and Quality Evaluation

According to the recommended meta-analysis method for observational epidemiological studies, and according to the PRISMA guideline, 4 English databases, including PubMed, Web of Science, Embase, and Cochrane Library, were searched from inception until December 2021. All cohort studies, prospective cohort studies, or case–control studies evaluating the relationship between serum ferritin levels and NAFLD were retrieved from these databases.

#### Inclusion Criteria:

Studies conducted in humans; studies examining the relationship between serum ferritin levels and NAFLD; prospective cohort studies, case–control studies, and cohort studies; studies with available data on relevant indicators such as serum ferritin levels, odds ratio (OR), and 95% CI, and studies conducted among individuals aged ≥18 years were included in the analysis.

#### Exclusion Criteria:

Studies based on animals; meetings, letters, case reports, reviews, meta-analyses, or systematic evaluations; duplicate or unrelated studies; studies lacking complete information and data; and studies with poor quality [the Newcastle–Ottawa Quality Assessment Scale (NOS) score of <5] were excluded.

The search strategy of PubMed is as follows: (“Hyperferritinemia”[MeSH Terms] OR (“Hyperferritinemias”[Title/Abstract] OR “raised serum ferritin”[Title/Abstract] OR “serum ferritin raised”[Title/Abstract] OR “elevated serum ferritin”[Title/Abstract] OR “serum ferritin elevated”[Title/Abstract] OR “Ultrahyperferritinemia”[Title/Abstract] OR “dysmetabolic hyperferritinemia”[Title/Abstract] OR ((“Hyperferritinemia”[MeSH Terms] OR “Hyperferritinemia”[Title/Abstract] AND “Dysmetabolic”[Title/Abstract]))) AND (“Non-alcoholic Fatty Liver Disease”[MeSH Terms] OR (“Non-alcoholic Fatty Liver Disease”[Title/Abstract] OR “NAFLD”[Title/Abstract] OR “nonalcoholic fatty liver disease”[Title/Abstract] OR “fatty liver nonalcoholic”[Title/Abstract] OR ((“fatty liver”[MeSH Terms] OR (“Fatty”[Title/Abstract] AND “Liver”[Title/Abstract]) OR “fatty liver”[Title/Abstract] OR (“Fatty”[Title/Abstract] AND “Livers”[Title/Abstract]) OR “fatty livers”[Title/Abstract]) AND “Nonalcoholic”[Title/Abstract]) OR “liver nonalcoholic fatty”[Title/Abstract] OR ((“Liver”[MeSH Terms] OR “Liver”[Title/Abstract] OR “Livers”[Title/Abstract] OR “livers”[Title/Abstract]) AND “nonalcoholic fatty”[Title/Abstract]) OR “nonalcoholic fatty liver”[Title/Abstract] OR “nonalcoholic fatty livers”[Title/Abstract] OR “nonalcoholic steatohepatitis”[Title/Abstract] OR ((“fatty liver”[MeSH Terms] OR (“Fatty”[Title/Abstract] AND “Liver”[Title/Abstract]) OR (“fatty liver”[Title/Abstract]) AND “Nonalcoholic”[Title/Abstract]) OR “steatohepatitis nonalcoholic”[Title/Abstract])).

### Quality Evaluation

The quality of the included literature was assessed using NOS. If the score was higher, the quality of the literature was higher. The NOS score ≥5 was considered as high-quality studies.

### Data Extraction

Two researchers independently screened the relevant literature according to the inclusion and exclusion criteria. The researchers discussed and solved any disagreements on which the literature should be included; however, if no consensus was reached, the decision was made after consulting a third person. The following data were extracted from the final included studies: the name of the first author, year of publication, country of study participants, type of study mean age, sex, total number of study population, number of people included in the NASH and no-NASH groups, number of people included in the NAFLD and no-NAFLD groups, methods of diagnosing NAFLD, NAFLD grading (if provided), mean and SD of the outcome index or effect size estimates (OR) and 95% CI, and the parameters adjusted in the study.

### Statistical Analysis

In this meta-analysis, NAFLD was considered as the outcome index, and the standardized mean difference, OR value, and 95% CI of serum ferritin were considered as the effect indexes. The chi-square test (*α* = 0.1) was used to analyze heterogeneity among the included studies; if *P* < .1 or *I*2 > 50% indicated a significant heterogeneity among the results of the studies, the random-effects model was chosen for the meta-analysis. Subsequently, a subgroup analysis was conducted according to the region of the participants, total number of participants, and methods of diagnosing NAFLD to eliminate the influence of confounding factors. A sensitivity analysis was then performed to specifically exclude 1 or several studies and to evaluate the stability of the meta-analysis results.

### Assessment of Publication Bias

Publication bias in the literature was determined using Begg’s and Egger’s tests; the included studies were considered to have no publication bias if the *P*-value of the Begg’s and Egger’s tests was >.05. If a conflict was observed between the *P*-values of the Begg’s test and Egger’s test, the *P*-values of the Egger’s test were used as a basis to determine the presence or absence of a publication bias, and the data were analyzed using the Stata 15.1 software.

## Results

### Screening Literature Results

**Figure 1.**
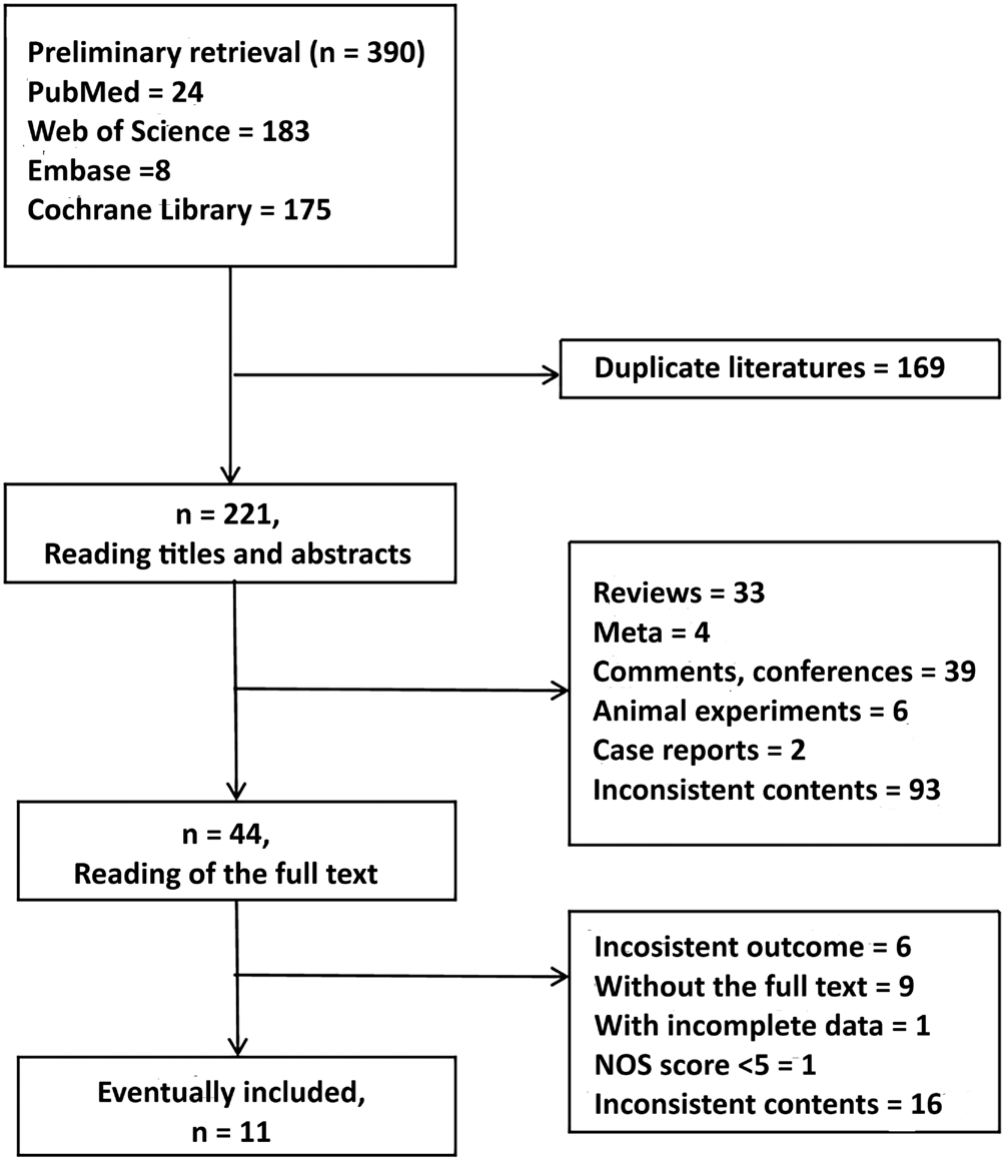
Flowchart of the literature screening process.

A total of 390 related literatures were obtained through a preliminary analysis. All literatures were imported into EndNote X9, while 169 duplicate literatures were excluded. Further reading the titles or abstracts, 177 papers were excluded, including 33 reviews, 4 meta-analyses, 39 comments and conferences, 6 animal experiments, 2 case reports, and 93 irrelevant studies. After carefully reading the full text of the remaining 44 papers, 33 papers were removed, including 6 with inconsistent outcome indicators, 9 with no available original text, 16 with inconsistent content, 1 with incomplete data, and 1 with an NOS score of <5; 11 studies were eventually included.^12,14-22^ The specific screening process is shown in Figure 1.

### Basic Features of the Included Literatures

The following data were extracted from the original studies (Table 1): name of the first author, year of publication, country of study participants, type of study, mean age, sex, total number of study population, number of people included in the NASH and no-NASH groups, number of people included in the NAFLD and no-NAFLD groups, methods of diagnosing NAFLD, NAFLD grading (if provided), mean and SD of the outcome index or effect size estimates (OR) and 95% CI, and parameters adjusted in the study.

Non-alcoholic fatty liver disease was diagnosed using abdominal ultrasound in 5 studies, liver biopsy in 4 studies, NAFLD liver fat score, hepatic and steatosis index (HSI) score in 1 study, and 3.5-MHz transducer in 1 study.

Among the 11 studies, 9 examined the relationship between serum ferritin levels and NAFLD^12,14,16,19-23^ and 6 examined the relationship between serum ferritin level and the risk of NAFLD.^12,14-18^

### Quality Evaluation of the Included Literatures

The quality of the included literature was assessed based on the NOS scale, with the case–control studies obtaining a score of >5 (Table 2). This suggests that the quality of the included literature was higher. The higher the score, the higher is the quality of the literature.

### Comparison of the Serum Ferritin Levels Between Non-alcoholic Fatty Liver Disease and Without Non-alcoholic Fatty Liver Disease Groups

**Figure 2.**
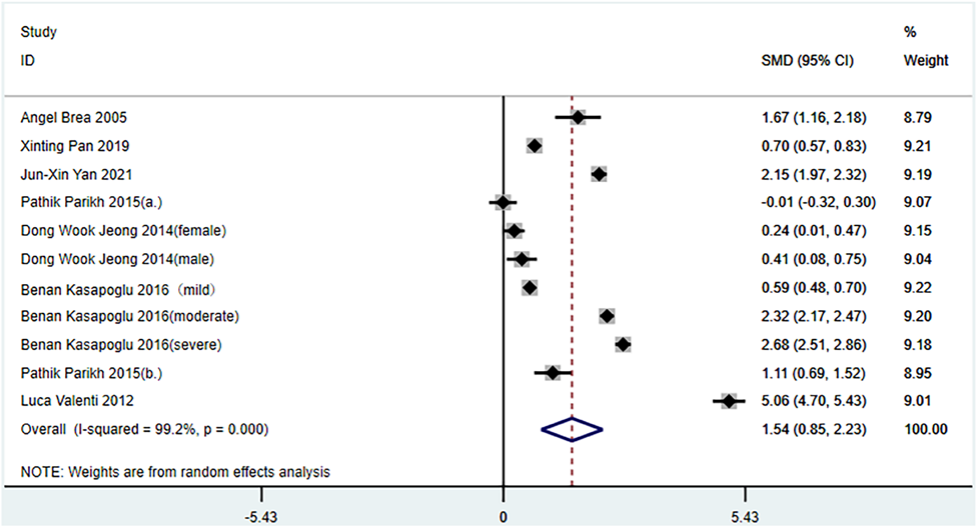
Comparison of serum ferritin levels between non-alcoholic fatty liver disease (NAFLD) and no-NAFLD groups. (a) Non-alcoholic fatty liver disease was diagnosed using abdominal ultrasound; (b) NAFLD was diagnosed using liver viability; mild/moderate/severe: NAFLD was graded using liver biopsy.

Seven studies compared the differences between NAFLD and non-NAFLD groups.^14,16,18-22^ After testing for heterogeneity (*I* ^2^ = 99.2%, *P *< .1), strong heterogeneity between the selected studies was observed; thus, a random-effects model was used for meta-analysis (Figure 2). The meta-analysis showed that the serum ferritin levels in the NAFLD group were significantly higher than those in the non-NAFLD group (1.54 ng/mL, 95% CI: 0.85-2.23*, P *< .001).

Publication bias was evaluated using the Egger’s test (*P* = .994), which suggested that there was no publication bias among these studies.

### Relationships Between Serum Ferritin Levels and Risk of Non-alcoholic Fatty Liver Disease

Four studies were included in the analysis.^14-17^ The meta-analysis showed that the heterogeneity was strong among all studies; therefore, a random-effects model was used for meta-analysis.

**Figure 3.**
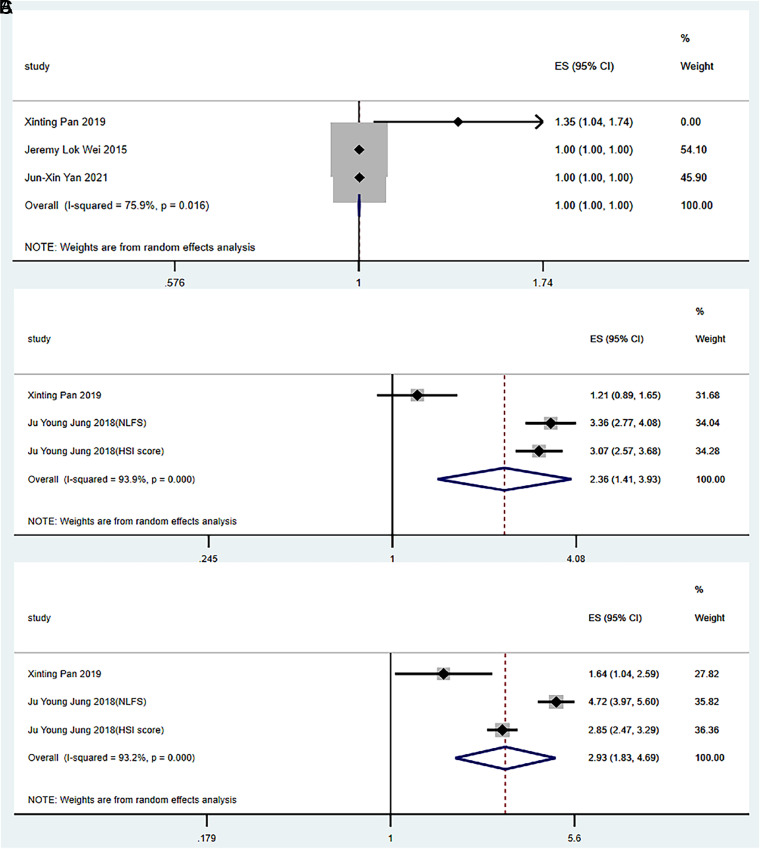
Relationships between serum ferritin levels and the risk of non-alcoholic fatty liver disease (NAFLD) (without adjustment). (A) General population, (B) male, and (C) female. HSI score, NAFLD diagnosed using the hepatic steatosis index; NLFS, NAFLD diagnosed using the hepatic fat score.

**Figure 4.**
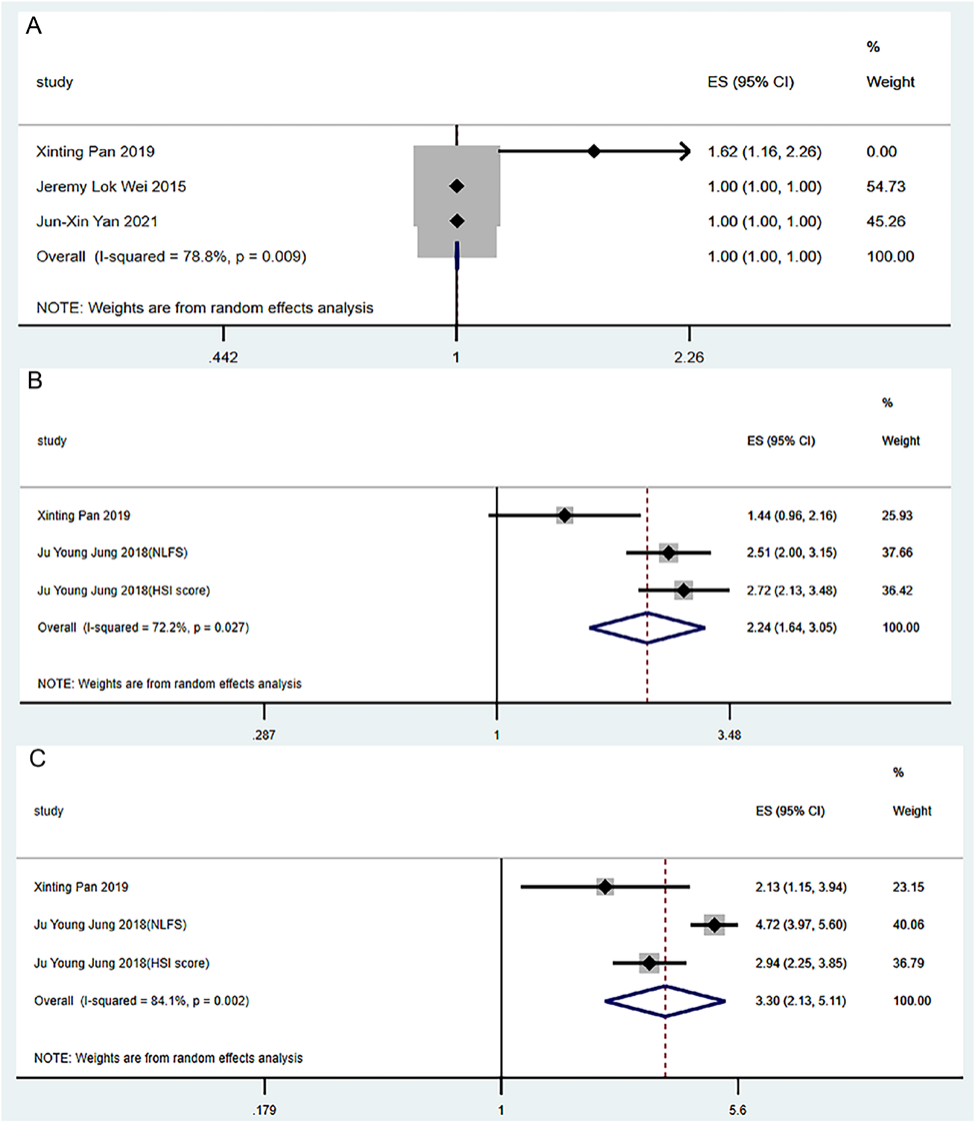
Relationships between serum ferritin levels and the risk of NAFLD (after adjustment). (A) General population, (B) male, and (C) female. HSI score, NAFLD diagnosed using the hepatic steatosis index; NLFS, NAFLD diagnosed using the hepatic fat score.

For the general population (without gender discrimination), before and after adjusting the parameters, no correlation was observed between serum ferritin levels and the risk of NAFLD (OR = 1.00, 95% CI: 1.00-1.00, *P* = 0.056; OR = 1.00, 95% CI: 1.00-1.00, *P* = 0.206) (Figures 3A and 4A).

The random-effects model results showed that serum ferritin levels were significantly associated with the risk of NAFLD in men and women (OR = 2.36, 95% CI: 1.41-3.93, *P *= .001; OR = 2.93, 95% CI: 1.83-4.69, *P *< .001) (Figure 3B and 3C) before adjusting the parameters.

After adjusting for the parameters, serum ferritin levels were significantly associated with the risk of NAFLD in men and women (OR = 2.24, 95% CI: 1.64-3.05, *P *< .001; OR = 3.30, 95% CI: 2.13-5.11, *P *< .001) (Figure 4B and 4C).

Publication bias before and after adjusting the parameters was assessed using the Egger’s test; the *P* values were .236 before adjusting and .052 after adjusting these parameters, which suggested that there was no publication bias among these studies.

### Meta-analysis of Serum Ferritin and Non-alcoholic Steatohepatitis

#### Comparison of Serum Ferritin Levels Between Non-alcoholic Steatohepatitis and Without Non-alcoholic Steatohepatitis Groups:

**Figure 5.**
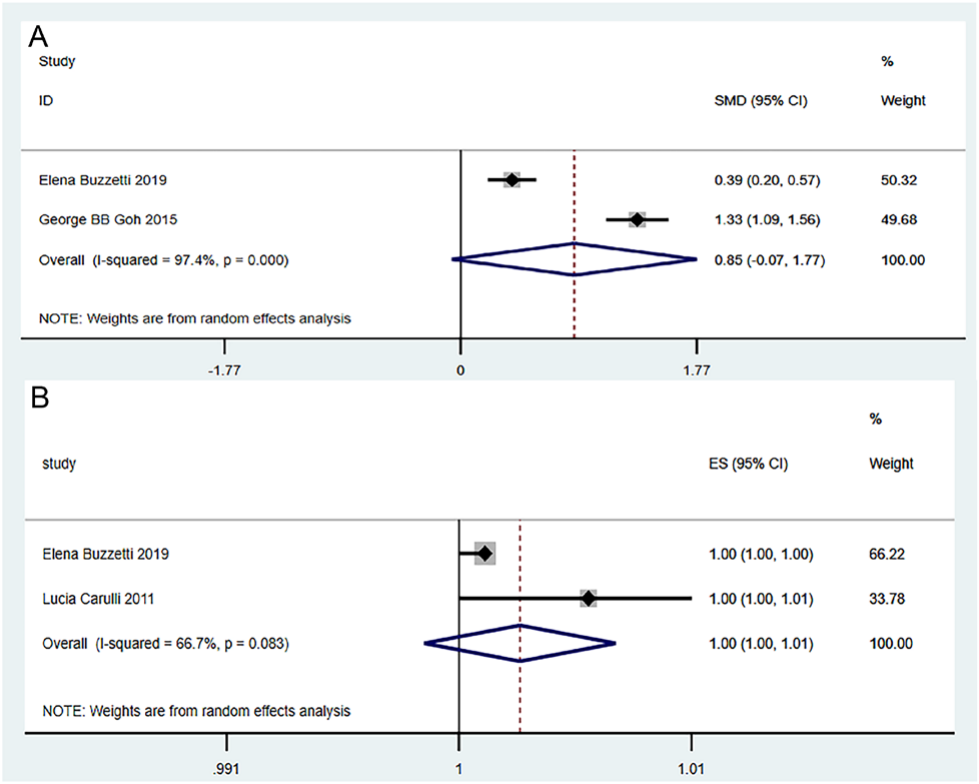
Meta-analysis of serum ferritin and the risks of non-alcoholic steatohepatitis.

Two studies were included.^12,23^ After testing for heterogeneity (*I* ^2^ = 97.4%, and *P *< .1), a strong heterogeneity was observed between the selected literatures. Thus, a random-effects model was selected for meta-analysis. The meta-analysis showed no significant difference in the serum ferritin levels between the NASH and non-NASH groups (0.85 ng/mL, 95% CI: 0.07-1.17,* P* = .070) (Figure 5A).

#### Relationships Between Serum Ferritin Levels and the Risk of Non-alcoholic Steatohepatitis:

Two studies were included.^12,18^ The meta-analysis results showed that heterogeneity existed among all included studies, and the random-effects model was selected for meta-analysis. Results showed that serum ferritin levels were not associated with the risk of NASH (OR = 1.00, 95% CI: 1.10-1.01, *P* = 0.213) (Figure 5B).

## Discussion

In the present study, a meta-analysis was conducted to confirm that the serum ferritin levels were significantly increased in patients with NAFLD and serum ferritin levels were correlated with the risk of NAFLD in both male and female populations. Yang et al^24^ reported that serum ferritin levels were increased in American adult NAFLD patients, while this correlation changed significantly with different gender and age. Du et al^25^ found that the serum ferritin levels in NASH patients were higher than those in no-NASH patients. However, no significant correlation was found between serum ferritin levels and NASH in the present study, which may be related to the age, gender, and geographical location of the included participants.

Although several studies have reported that the serum ferritin is related to NAFLD, the specific mechanism remains unclear; Serum ferritin levels and ferritin/hepcidin ratio are significantly correlated with NAFLD in the Chinese population.^14^ Yan et al^16^ reported that the serum ferritin levels were an independent risk factor for NAFLD in middle-aged and older patients with type 2 diabetes. Jeong et al^21^ found that serum ferritin levels were closely related to the severity of NAFLD, and the risk of NAFLD significantly increased with an increase in serum ferritin quartile levels in the male population. A dose–effect correlation was observed between serum ferritin levels and the occurrence of NAFLD. With the increase in ferritin levels, the blood glucose and insulin levels gradually increase, further aggravating insulin resistance, which is a potential mediating factor in the relationship between serum ferritin and NAFLD, and obesity, which is one of the risk factors for NAFLD. With the accumulation of serum ferritin levels in the body, body mass index (BMI) and waist circumference also showed a significant increase.^17^

Non-alcoholic fatty liver disease is a complex chronic liver disease that is usually accompanied by other comorbidities (such as obesity, insulin resistance, and metabolic syndrome), which may lead to NASH and HCC.^26^ Major manifestations of NAFLD are insulin resistance, lipid accumulation, and chronic inflammation.^27^ Insulin resistance and lipid accumulation in the liver lead to a series of mitochondrial dysfunctions and lipid peroxidation, thus damaging the mitochondria and liver cells, ultimately leading to fibrosis and cirrhosis.^28^ Liver inflammation can be caused by the accumulation of lithium toxic metabolites, oxidation, endoplasmic reticulum stress, tissue hypoxia, and endothelial cell dysfunction.^29^ Some studies have shown that intestinal microbes play a key role in the pathogenesis of NAFLD. Unhealthy diet and drugs can lead to ecological imbalance of the intestinal microbiota, increased intestinal mucosal permeability, and excessive toxicity or pro-inflammatory molecules, which negatively affect the liver physiology. Dysfunctional livers are unable to effectively control the intestinal microbiota through bile acids and other microbial regulatory factors, resulting in intestinal ecological imbalance and intestinal barrier dysfunction. This vicious cycle has a significant impact on the liver, intestine, and human health.^30^ Recent studies have shown that cellular aging mechanisms may be involved in the progression of NAFLD to severe NASH and HCC, which mainly manifested as mitochondrial dysfunction, excessive reactive oxygen species production, reduced NAD+, increased AmP-activated protein kinase activity, and biological energy imbalance, which further activate and promote hepatocellular senescence.^26^

Adult NAFLD patients often experience changes in serum iron metabolism, manifested by increased serum ferritin levels and normal transferrin saturation, which is known as the metabolic abnormal iron load syndrome.^25^ Iron accumulation in adult patients with NAFLD is due to impaired iron mobilization in the liver cells and Kupffer cells.^31^ Serum ferritin, a major marker of liver iron storage, positively correlated with liver fat accumulation. Intestinal bacteria and body iron status play an important role in the pathological mechanism of NAFLD. Ferritin is negatively correlated with Pasteurellaceae, Leuconostocaceae, and Micrococcaceae families, while ferritin is positively correlated with Bacteroides and Prevotella species. Ferritin-related bacterial families are strongly correlated with liver iron-related genes, and such iron metabolism-related microbiome characteristics may be correlated with liver fat accumulation through liver glucose metabolism.^32^ Serum ferritin elevation in NAFLD patients is mainly related to chronic liver inflammation, β-cell dysfunction, and insulin resistance.^33^ Kim et al^13^ found that serum ferritin could generate hydroxyl-free radicals through an iron-catalyzed peroxide reaction, thus increasing tumor necrosis factor and enhancing oxidative stress. Second, increased serum ferritin levels may reduce insulin sensitivity, which can increase the accumulation of free fatty acids through de novo synthesis of liver fat and decomposition of liver adipose tissue, directly leading to liver dysfunction.

As a pathologic type with progressive risk of NAFLD disease, 38% of NASH patients can progress to fibrosis within 5 years, and approximately 20% can progress to cirrhosis within 10 years.^34^ Current studies have attempted to explore the role of serum ferritin in patients with NAFLD, especially its association with NASH or fibrosis, as a non-invasive marker. Kowdley et al^35^ found that serum ferritin was positively correlated with NASH and/or fibrosis, whereas Kowdley et al^35^ reported the opposite result. More interestingly, serum ferritin levels were associated with an increased risk of developing hepatic steatosis, but not with NASH, which is not independently related to liver fibrosis.^12^ Goh et al^23^ believed that serum ferritin was not an accurate predictor of NASH. To further improve the accuracy, they combined serum ferritin, aspartate aminotransferase, BMI, platelet count, diabetes, and hypertension history to establish NASH predictive scoring (NPS) models. The NPS model can facilitate the screening of patients who need to undergo liver biopsy and even help hepatologists to further evaluate the patient’s condition, which is conducive to the formulation of drug treatment plans.

This study has some limitations. First, all included studies were case–control studies, which could not explain the causal relationship between serum ferritin levels and NAFLD. Second, the gold standard for the diagnosis of NAFLD is liver pathological tissue biopsy; however, in the included studies, different methods were used for diagnosing NAFLD, such as liver ultrasound or liver biopsy, which may lead to heterogeneity in the diagnosis of NAFLD and NASH. Finally, there is a hierarchy among the populations included in this study, which may also have a certain influence on the mean serum ferritin level.

In conclusion, serum ferritin levels in NAFLD patients are significantly increased and are closely related to the risk of NAFLD. Ferritin together with other metabolic and biochemical parameters may improve the performance of non-invasive scores for NAFLD, and iron depletion still represents an attractive therapeutic target to prevent the progression of liver damage.

## Figures and Tables

**Table 1.** Basic Features of the Included Literatures

First Author	Country	Type of Study	Number (Male, Female), Mean Age)	Definition of NAFLD			Adjusted Parameters
			Total	NAFLD No NAFLD	NASH No NASH		
Pan et al^14^	China	Case–control study	972 (659, 313), 48	482 (328, 154), 48	—	Liver ultrasonography	Waistline, SBP, DBP, FPG, TG, TC, HDL-C, ALT, AST, γ-GT, and CRP
				490 (331, 159), 48			
Wei et al^15^	Hong Kong	Case–control study	991 (382, 277), 48	262 (141, 121), 51	—	Whole-body 3.0T scanner	Age, gender, BMI, SBP, DBP, waist circumference, creatinine, fasting glucose, HbA1c, TC, ferritin, HOMA-IR, TG, LDL, HDL, and PNPLA3
				729 (382, 347), 49			
Yan et al^16^	China	Case–control study	805, 59.78	427, 61	—	Liver ultrasonography	Age, duration, BMI, Hb, MCHC, VB12, FA, AST, albumin, TBil, TP, DBil, UA, TC, SBP, HDL, LDH, RBC count, HbA1c, FPG, and DBP
				378, 58			
Jung et al^17^	Korean	Case–control study	25 597 (9615, 15 982), >20	—	—	NAFLD liver fat score (2) hepatic steatosis index score	Age, SBP, BMI, FPG, diabetes mellitus, TC, daily alcohol intake, smoking history, and PA
Buzzetti et al^12^	European	Case–control study	468 (260, 177), 47	—	247 (148, 99), 50	Liver biopsy	—
					221 (143, 78), 45		
Valenti et al^18^	Italian	Case–control study	487 (381, 105), 49	216 (167, 49), 49	—	Biopsy proven	—
				271 (215, 56), 45			
Brea et al^19^	USA	Case–control study	80 (40, 40), 53	40 (20, 20), 53	—	Liver ultrasonography	—
				40 (20, 20), 51			
Kasapoglu et al^20^	Turkey	Case–control study	2058 (1563, 495), 50	473 (380, 93), 49	—	(Mild) liver ultrasonography	—
				982 (792, 190), 45			
Kasapoglu et al^20^	Turkey	Case–control study	2058 (1563, 495), 50	363 (273, 90), 50	—	(Moderate) liver ultrasonography	—
				982 (792, 190), 45			
Kasapoğlu et al^20^	Turkey	Case–control study	2058 (1563, 495), 50	240 (118, 122), 51	—	(Severe) liver ultrasonography	—
				982 (792, 190), 45			
Jeong et al^21^	Korean	Case–control study	558 (295, 163)	172 (132/40), 42	—	3.5-MHz transducer	—
				386 (163/22)3, 40			
Parikh et al^22^	India	Case–control study	300 (165, 190), 18	250 (88, 162), 44	—	250 liver ultrasonography and 55 liver biopsy	—
				55 (40, 15), 42			
				50 (37, 13), 41			
Goh et al^23^	USA	Case–control study	405 (178, 226), 48	405 (178, 226), 48	291 (126, 164), 49	Biopsy proven	—
					114 (52, 62), 46		

ALT, alanine aminotransferase; AST, aspartate aminotransferase; BMI, body mass index; CRP, C-reactive protein; DBil, direct bilirubin; DBP, diastolic blood pressure; FA, fatty acid; FPG, fasting blood glucose; Hb, hemoglobin; MCHC, Mean corpuscular hemoglobin concentration; HbA1C, glycosylated hemoglobin; HDL-C, high-density lipoprotein cholesterol; HOMA-IR, insulin resistance index; LDH, lactate dehydrogenase; LDL, low-density lipoprotein; NAFLD, non-alcoholic fatty liver disease; NASH, non-alcoholic steatohepatitis; PA, physical activity; PNPLA3, patatin-like phospholipase domain-containing protein 3; RBC, red blood cell; SBP, systolic blood pressure; TBil, total bilirubin; TC, total cholesterol; TG, triglyceride; TP, total protein; UA, uric acid; VB12, vitamin B12; γ-GT, γ-glutamyltranspeptidase.

**Table 2.** Quality Evaluation of Case–Control Studies

Study	Selection				Comparability Adjusted Confound Factors	Exposure			Total
	Case Definition	Case Representativeness	Selection of Controls	Definition of Control		Ascertainment of Exposure	Ascertainment of Cases and Controls	No Response Rate	
Pan et al^14^	1	1	1	1	2	1	1	0	7
Wei et al^15^	1	1	0	1	2	1	1	0	7
Yan et al^16^	1	1	1	1	2	1	1	0	8
Jung et al^17^	1	1	0	1	2	1	1	0	7
Buzzetti et al^12^	1	1	0	1	2	1	1	0	7
Valenti et al^18^	1	1	1	1	1	1	1	0	7
Brea et al^19^	1	1	1	1	0	1	1	0	6
Kasapoglu et al^20^	1	1	0	1	1	1	1	0	6
Jeong et al^21^	1	1	0	1	1	1	1	0	6
Parikh et al^22^	1	1	1	1	0	1	1	0	6
Goh et al^23^	1	1	0	1	2	1	1	0	7
